# Pressure Injury Surveillance and Prevention in Australia: Monash Partners Capacity Building Framework

**DOI:** 10.3389/fpubh.2021.634669

**Published:** 2021-10-28

**Authors:** Victoria Team, Angela Jones, Helena Teede, Carolina D. Weller

**Affiliations:** ^1^Monash Nursing and Midwifery, Faculty of Medicine, Nursing and Health Sciences, Monash University, Melbourne, VIC, Australia; ^2^Monash Partners Academic Health Science Centre, Clayton, VIC, Australia

**Keywords:** Australia, acute health care services, capacity building framework, collaboration, consumer involvement, hospital-acquired pressure injury, research-to-practise gap

## Abstract

A hospital-acquired pressure injury (HAPI) is a common complication across the globe. The severity of HAPI ranges from skin redness and no skin breakdown to full skin and tissue loss, exposing the tendons and bones. HAPI can significantly impact the quality of life. In addition to the human cost, this injury carries a high economic burden with the cost of treatment far outweighing the preventative measures. The HAPI rates are a key indicator of health services performance. Globally, healthcare services aim to reduce its incidence. In Australia, the federal health minister has prioritised the need for improvement in HAPI surveillance and prevention. Capacity building is vital to optimise pressure injury (PI) surveillance and prevention in acute care services. In this perspective article, we provide a framework for capacity building to optimise HAPI prevention and surveillance in a large cross-sector collaborative partnership in Australia. This framework comprises six key action areas in capacity building to optimise the HAPI outcomes, such as research, organisational development, workforce development, leadership, collaboration, and consumer involvement.

## Introduction

A pressure injury (PI) is defined as a localised injury to the skin and/or underlying tissue often situated over a bony prominence, often caused as a result of prolonged pressure (1). The main constructs in predicting the risk of PI are pressure, shear and friction, and tissue tolerance (2). The patient-related factors, such as poor nutrition and chronic illness, which impact tissue tolerance increases the PI development risk. A PI acquired in a hospital setting is referred to as hospital-acquired pressure injury (HAPI). Admission type, surgical interventions, medications, the length of hospital stay, and hospital environments, such as nurse staffing, unit type, and nursing workload have all been identified as potential impacts on the HAPI development (2). Depending on the severity of the injury, PI ranges from skin redness to full skin and tissue loss, exposing the tendons and bones (1). There are four stages of PI, such as stage I–non-blanchable erythema, stage II–partial thickness skin loss, stage III–full thickness skin loss, and stage IV–partial thickness tissue loss (1). The cases, where the depth of the injury is unknown, are classified as either unstageable PI due to the presence of eschar or slough limiting the ability of the assessor to stage or suspected deep tissue injury if a localised skin area is of discoloured purple or maroon colours (1). PI-related pain, wound exudate and odour, reduced mobility, and lack of independence may significantly impact the quality of life of a person (3–5). PI may be complicated with osteomyelitis and sepsis (6–8). According to the latest international one-day point-prevalence study of HAPI in 90 countries, the overall prevalence of HAPI was 26.6% (95% CI 25.9–27.3); and intensive care unit (ICU)-acquired prevalence was 16.2% (95% CI 15.6–16.8) (9).

## The Need For Capacity Building to Prevent Hapi

Hospital-acquired pressure injury results in a high economic burden in healthcare, primarily due to increased hospital stay and management of hospital-acquired complications (10). A cost-effectiveness analysis conducted by the US health economists reported that HAPI prevention in the hospitalised patients is cost-effective; and remains the highest value alternative, although the investment in technology may be required (11). Although the cost-savings of different HAPI prevention interventions may vary (12, 13); in general, HAPIs cost more to treat than to prevent (10, 11, 14). HAPI is considered an indicator of the quality of care in acute care services (15, 16). This common hospital-acquired complication is largely preventable or deemed a never event (17). The clinical practise guidelines (1) provide the best evidence on HAPI preventive methods. However, the studies (18, 19) report difficulties in translating the best evidence into clinical practise. Many patients do not receive evidence-based HAPI preventive care, and there are reported gaps between the planned and implemented HAPI preventive strategies (19, 20). In Australia and globally, the studies have shown that the knowledge and skills of clinicians in HAPI prevention are suboptimal (21–25). Rapid population ageing in Australia and globally coupled with the increased prevalence of chronic illnesses that requires hospital admissions has an impact on the acute health service capacity to prevent and manage HAPI (26).

## Capacity Building Strategies

The WHO [(27), p. 341] defined capacity building as “the development of knowledge, skills, commitment, structures, systems, and leadership to enable effective health promotion.” This development requires three-levels of action: “the advancement of knowledge and skills among the practitioners; the expansion of support and infrastructure for health promotion in the organisations; and the development of cohesiveness and partnerships for health in communities” (p. 341). Globally, the capacity building interventions have been reported to enhance the desired capacity building outcomes both on the individual and public health system levels (28).

Although the complex multicomponent HAPI prevention programs have been implemented in the past, these have been conducted in the individual hospitals (29) and ICUs (30). The main components of these programs include preparatory activities, such as baseline prevalence and incidence surveys, assessments of the knowledge of clinicians, and review of the existing policies and practises; implementation of the evidence-based practises; workforce education, and improved clinical monitoring and feedback (29). In ICUs, these multicomponent HAPI prevention programs have been conducted as “before and after” the research studies or quality improvement projects (30). A framework of quality improvement interventions to implement the evidence-based practises for HAPI prevention developed by Padula and associates (31) reported on the leadership initiatives and teamwork; staff education, training, and performance assessment; clinical practise performance improvement, and information technology interventions, such as data tracking and the use of electronic health record for risk assessment. The study aimed to provide a practical example of capacity building to optimise HAPI prevention and surveillance across a network of seven acute health services within a large cross-sector collaborative partnership in Australia.

## Ethical Considerations

This perspective article is informed by the information derived from two Monash Partners research projects approved by the Human Research Ethics Committees at Monash Health (NMA/ERM Reference Number: 58533; Monash Health Ref: RES-19-0000755L-58533) and the Alfred Health (Project Number: 62256; Local Reference: Project 252/20), Australia.

## The Proposed Capacity Building Framework

**Figure 1:**
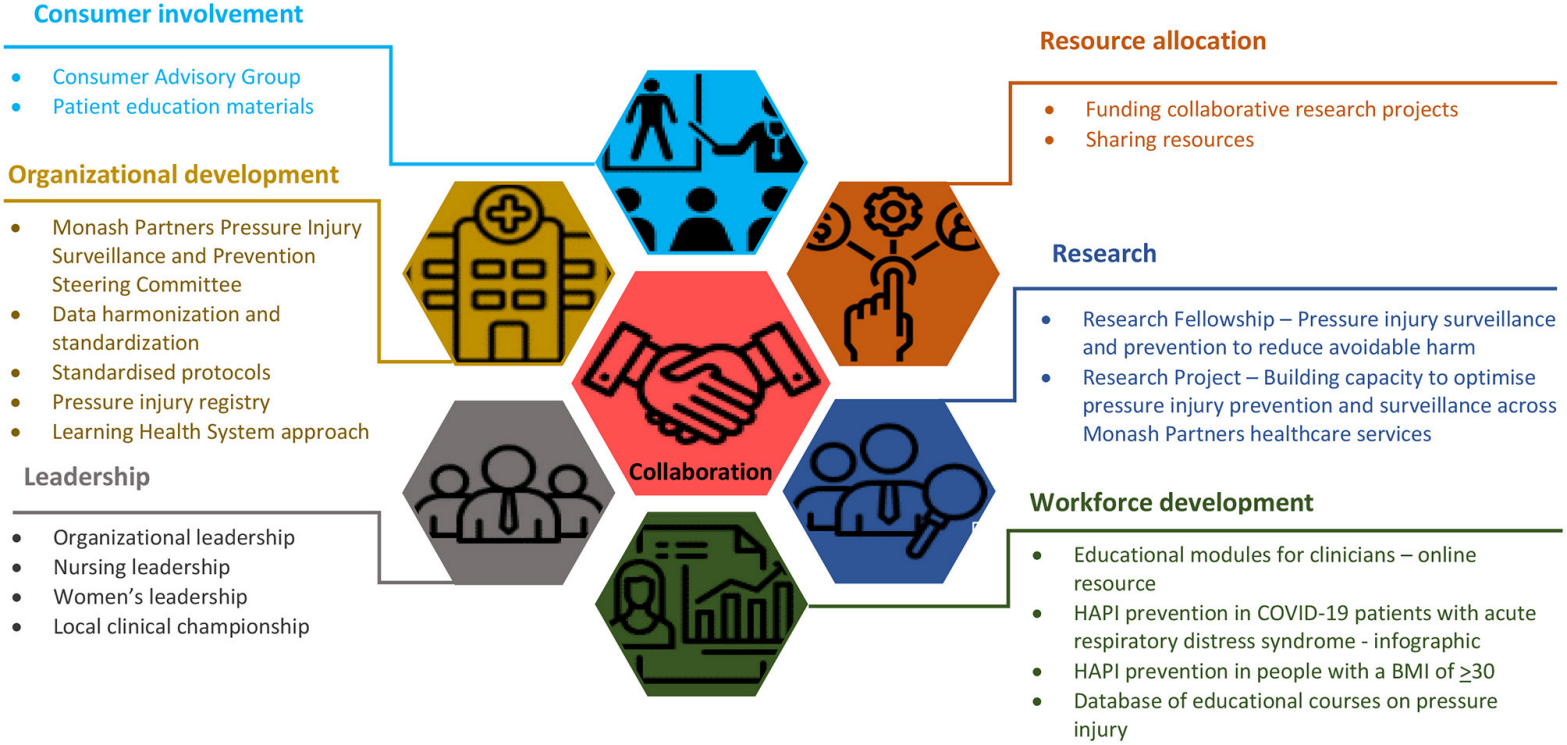
Pressure injury surveillance and prevention. Monash Partners Capacity Building Framework.

To inform PI capacity building for preventing and managing HAPI across the Monash Partners network we utilised a Framework for Building Capacity to Improve Health developed by the New South Wales Health Department (32). This framework consists of the five key action areas in capacity building: organisational change, workforce development, resource allocation, partnerships, and leadership (32). We adapted this framework by adding two other major action areas—resource allocation and consumer involvement (Figure 1).

Collaboration is the central component of our framework because capacity building to optimise HAPI surveillance and prevention is a complex task requiring collaboration on multiple levels, such as interprofessional collaboration and collaboration with partnering health services, professional associations, consumer groups, and research centres nationally and internationally. A collaborative approach allows for gathering uncertainties, setting research priorities, facilitating implementation, and promoting the faster translation of findings into clinical practise in wound care (33).

Financial resource allocation is a second vital component of the proposed capacity building framework. The findings of a recent cost-effectiveness analysis reported the need for health systems to invest in the quality-improvement infrastructure (11). The cost of resources allocated for HAPI prevention for all hospitalised patients would balance and outweigh the cost of management of stage 3, 4, or unstageable HAPI (11). The collaborative approaches that enable sharing resources across the partnering health services and creating uniform resources may reduce the cost of the preventive strategies.

Collaborative research enables and enhances the utilisation of research findings in clinical practise and informs an evidence base relevant to the end users—clinicians (34). These outcomes are achieved through the co-production of research (35), which facilitates rapid translation of the latest evidence into the clinical practise and reduces the evidence-practise gap as well as research waste (36). The priority issues for HAPI research in Australia include the strategies for assessing skin and tissues; consensus on the outcome measures; and economic cost of HAPI prevention and management strategies among others (37).

Leadership is a critical determinant of success in complex, cross-sector collaborations that aim to translate evidence into practise (37). Moreover, effective leadership correlates with the quality of care and improved patient outcomes (38). Advocacy from system leadership and local unit clinical championship in HAPI prevention coupled with the quality improvement components could optimise the successful implementation of the evidence-based guidelines in HAPI prevention (38).

Effective evidence informed interventions rely on high-quality data. However, the available statistical information on HAPI reported by the health services is difficult to compare because of the differences in surveillance and reporting system across the countries, states within one country, and even individual health services (15, 39, 40). The lack of consistency and uniformity in the current reporting of HAPI in Australia leads to inaccurate data interpretation and hinders the improvement in accuracy of HAPI identification and prevention (15). The high-quality HAPI incidence data allow to benchmark performance with other hospitals and reduce the incidence of preventable HAPI. The need to collect high-quality HAPI incidence data over time has been highlighted by Australian researchers (15, 39). The organisational development approaches, such as data standardisation and wound registry, may attend to this need (26).

Workforce capacity building is a vital strategy in accelerating the research translation in clinical practise (36). The knowledge and skills of clinicians in HAPI prevention, management, and reporting (40) require ongoing development to facilitate faster translation of the best evidence into clinical practise (41). The impact of education of the healthcare professionals on the prevention of HAPI is under research, and there is no evidence that individual educational interventions for healthcare professionals about HAPI prevention are effective in reducing the HAPI incidence and improving the knowledge of nurses of HAPI prevention (42). However, some complex multicomponent HAPI prevention programs containing educational components were found to be effective (30).

The voice of the consumer is central to the wound research and practise (26), and there is a need to move from the long-standing perception of patients as passive recipients of wound care to the new perception of them as individuals providing an important voice in the wound care and research (43). To date, there is a paucity of research reporting the role of consumers in optimising evidence-based management and prevention of HAPI (44). Developing effective partnerships among clinicians, researchers, and consumers is an effective approach in establishing patient-centred care (45, 46). We further describe Monash Partners implementation of the proposed capacity building framework, detailing the key aspects of this model.

## Monash Partners Implementation of the Proposed Capacity Building Framework

Monash Partners Academic Health Science Centre is a partnership among the 12 leading health services, research, and teaching organisations serving a population of almost 3 million Australians (www.monashpartners.org.au). The partner organisations include Alfred Health, Monash Health, Monash University, Cabrini Health, Epworth HealthCare, Eastern Health, Peninsula Health, Baker Institute, Burnet Institute, and Hudson Institute with La Trobe University, and Latrobe Regional Hospital as associate partners. Established in 2011, the purpose of Monash Partners is to “connect the researchers, clinicians, and the community to innovate for better health” (47). Monash Partners was accredited by the National Health and Medical Research Council (NHMRC) as a research translation centre. The Australian Health Research Alliance (AHRA), comprising all 10 NHMRC-accredited research translation centres (48), recognised the need to improve the patient outcomes and has established a national wound care special initiative, bringing together the clinicians, researchers, and consumers from acute health services, the community and aged care. In 2019, an AHRA steering committee proposed a National project aimed at addressing the challenges in wound care through an integrated, evidence-based approach (48). Monash Partners along with other NHMRC-accredited centres supports the integration of research, education, and clinical practise, overcoming the organisational silos and barriers to research translation (49), such as in HAPI prevention.

Our HAPI prevention team consists of the leading academic in the field, a competitively funded health services research fellow specialised in wound care, the Executive Director and Chief Operating Officer of Monash Partners, providing executive support, and the Monash Partners Pressure Injury Surveillance and Prevention Steering Committee. The Monash Partners Pressure Injury Surveillance and Prevention Steering Committee has been established with the aim of contributing to research design data collection and data analysis, and utilisation of the findings to improve HAPI prevention in the participating health services. This governance allows bringing together the researchers, clinicians, and consumers across the network and enables linking into a national network of wound care experts.

We work in close collaboration with our interstate partners. For example, together with the Brisbane Diamantina Health Partners, our Monash Partners team is co-designing a National Educational Framework in Wound Care. Monash Partners also collaborates with Wounds Australia—the peak National body for wound prevention and management; and has a representative from this association at the Monash Partners Steering Committee on Pressure Injury Surveillance and Prevention, which aims to enhance the current efforts in this area and link to a national collaboration around the wound management. Finally, we have existing and newly established international collaborations with the leading researchers from Canada, Ireland, United Kingdom, and Sweden. For example, we seek to adopt the best practises from the Swedish National Quality Registry of Ulcer Treatment, which helped to improve the wound healing outcomes and significantly reduced costs (50).

Monash Partners next step was allocating resources to the health services research projects. Since 2019, the two major research projects are being carried out by Monash Partners, which are supported by the Medical Research Future Fund (MRFF) as part of the Rapid Applied Research Translation program. A research fellow has undertaken a range of activities, such as an audit of exiting HAPI prevention activities; conducted a needs assessment in education and training in HAPI surveillance, prevention, and management across the Monash Partners acute care services; conducted a comparison of the three different HAPI data sources in one of the Monash Partners health services; scoped current education and training programs across Victoria and whether these are accredited; and analysed the availability and content analysis of HAPI information available on the health services websites across the state of Victoria, Australia. The current multicentre collaborative capacity building research project aimed to (1) map and compare existing HAPI data across four Monash Partners health services; (2) develop and pilot HAPI data harmonisation approach; (3) identify the alignment of the HAPI assessment tool/s and HAPI coding definitions; (4) standardise risk adjustment procedures to account for the differences in risk of HAPI development; (5) identify individual, organisational, and health system level barriers to integrate the HAPI assessment and care across the continuum; (6) develop, pilot, and evaluate the training modules for the clinicians and medical record coders to ensure accurate HAPI assessment, documentation, and coding; (7) establish and evaluate the cost-effectiveness of pilot HAPI clinical registry; and (8) evaluate the impact of the pilot HAPI clinical registry on the patient outcomes. The described research activities emerged from the existing partnerships; with new collaborations also created for these activities (51).

The discussed Monash Partners HAPI research projects are conducted by the nurses and in close collaboration with nurses. Although HAPI prevention is based on multidisciplinary teamwork (39), most preventive activities are undertaken by the nursing staff; and the importance of the leading role of nurses in HAPI prevention is well-recognised (40).

As part of organisational development, Monash Partners works to improve the HAPI data quality, harmonise HAPI data in collaborating health services, and establish a wound registry, providing a minimal dataset. A study conducted as part of the Research Fellowship involved a comparison of the three different HAPI data sources in one of the Monash Partners health services, to improve the accuracy and comparability of data (39). The findings from this study provided benchmark areas for the improvement in HAPI documenting and reporting. The discussed improvements in the HAPI data quality are in line with the AHRA data-driven healthcare improvement activities (52) and the Monash Partners Learning Health System approach to guide the evidence informed decision-making in healthcare delivery (53). The Learning Health Systems provide the necessary elements and create environments, enabling the clinicians to translate research into practise and the consumers to participate in research (54, 55). The trend to transform the healthcare services into a Learning Health System may shape the healthcare systems of the future (56). Monash Partners leads the Integrated Training and Education in Wound Care stream of the AHRA Wound Care Initiative and participates in the Coordinated Program of Research stream (48).

Monash Partners is committed to supporting the workforce development in HAPI surveillance and prevention in partnering with the health services. As part of the research fellowship project, interviews were conducted with the research, education, and clinical leads in HAPI prevention and management, who represent partnering organisations at Monash Partners Steering Committee. The data analysis indicated that although a variety of training courses on HAPI prevention and management are available across the partnering health services, they are lacking uniformity; some of them are informal; and, in some health services, there are waiting lists to attend these courses. The expressed educational needs included PI classification and staging, differential diagnostics with incontinence-associated dermatitis, HAPI prevention in people with a body mass index of 30 and over, HAPI prevention in prone positioned people, and HAPI reporting. To meet these needs, Monash Partners convened series of educational webinars for the healthcare workers delivered by the researchers and frontline clinicians in the field. Further, we developed a needs-oriented online educational resource (https://monashpartners.org.au/education-training-and-events/pressure-injury/) consisting of five modules to improve the knowledge and skills of clinicians in the field of HAPI prevention. In addition, we scoped available educational courses on HAPI prevention and management in Victoria, which will comprise part of the National database of educational courses on wound care.

As an emergency response to the COVID-19 outbreak in Melbourne, we have developed an infographic on HAPI prevention in the patients with COVID-19 with an acute respiratory distress syndrome in the prone position and communicated it to the partnering health services (57). The need to improve the skills of acute care clinicians in HAPI management emerges with the influx of COVID-19 aged care residents with PI detected on admission (58). Acquiring the knowledge and skills on HAPI prevention in patients with COVID-19 is critical, as many patients cannot be active participants in their care. In collaboration with Sydney Health Partners, we have organised a webinar on HAPI prevention in the prone positioned patients with COVID-19 with an acute respiratory distress syndrome. Frontline clinicians from both the states have shared their practises and experience in HAPI prevention in COVID-19 patients, such as the use of prophylactic dressings to protect the prone positioned patients from facial HAPI; the introduced functional teams that assist with repositioning the prone-positioned patients; virtual consultations with the wound clinical nurse consultants, and the use of personal protective equipment. Monash Partners has also developed an online educational module for healthcare professionals on HAPI prevention in prone-positioned patients with COVID-19.

At Monash Partners, we are building a strong partnership among the patients, their families, caregivers, researchers, and healthcare professionals through the Consumer Advisory Group convened in 2019. The Consumer Advisory Group representative is in the Project Advisory Committee and provides advice on all the steps of the building capacity to optimise PI prevention and surveillance across the Monash Partners healthcare services. In addition to the involvement of consumers in research, we involve them in the assessment and development of education materials. For example, we conducted a small-scale research project on the availability and content analysis of the online patient education materials on PI prevention in hospitals and health services in Melbourne Metropolitan and rural Victoria (59). We found that a greater proportion of hospitals did not have any of these materials publicly available, with private hospitals and metropolitan hospitals more likely to have materials available on their sites compared with the public and rural hospitals. The available materials contained accurate messages on the PI defining characteristics and risk factors for PIs, although many of these materials were not approved by a consumer group (59). We plan to develop a repository of patient education materials on PI prevention, which will be regularly updated and approved by the Consumer Advisory Group. This repository will be accessible to the health professionals from the partnering health services and available for distribution to their consumers.

## Barriers and Benefits of This Approach

The main challenge to the implementation of this complex capacity building framework is related to the lack of uniformity in HAPI definition, HAPI surveillance, and current HAPI protocols and prevention practises across the partnering services. Implementation is an ongoing process and at this stage, we scoped the available HAPI definitions and protocols and surveillance methods. Our next step is to involve stakeholders in planning on how to ensure the uniformity of the HAPI prevention protocols and practises and standardise the data collection process across the partnering services. After discussing the uniformity of educational resources with the health services leads, we decided that the newly developed uniform educational resources would complement the available resources rather than replace them.

A further barrier was workforce redirection to frontline clinical work since the beginning of the COVID-19 pandemic in Australia. The nurses' intensive frontline work and related burnout, periods of personal isolation and uncertainty, and home schooling their children during repeated lockdowns (60–62) shifted their focus away from the continuing professional development. Our recent interviews with the nurses also indicated that since the beginning of the pandemic, some regular HAPI preventive activities, such as interprofessional meetings and face-to-face skin integrity workshops, have been discontinued. Following the requirements of the recent COVID-19 safety plans for health services and the universities, our planned research and educational activities, such as in-person focus group discussions and face-to-face HAPI prevention workshops, were replaced with online meetings and webinars.

The introduced framework allows to ensure appropriate infrastructure exists to support HAPI education, prevention, and care throughout the continuum. In general, the collaborative approach allows us to overcome the silos across the partnering organisations, facilitate rapid implementation and reduce the research-to-practise gap (36). The collaborative research projects, shared educational materials and developed uniform educational resources, which means we can cut costs for collaborators. This cost saving capacity building approach is particularly important at the time of the latest pandemic when there is a need to prioritise resource stewardship (61).

## Concluding Comments

Theory-informed capacity building increases the likelihood of developed changes to be sustained and enables the health services to have a greater capacity in addressing future challenges (32). Enhanced capacity has the potential of reducing the gap among the evidence and practise, promoting problem solving, and achieving health gains (63). The collaborative approaches are suitable for the study of complex phenomena (34), such as a capacity building. We provided a practical example of capacity building to optimise HAPI prevention and surveillance; and shared our capacity building framework.

### Limitations

The main limitation of this perspective article lies in its descriptive nature. The descriptive studies have been criticised for their micro-level engagement that limits generalisability and provides anecdotal evidence (64). While we have outlined our implementation plan and the process of capacity building, we have not been able to report on the effectiveness of this approach that includes reduced incidence of HAPI across the Monash Partners acute care services.

## Ethics Statement

The studies involving human participants were reviewed and approved by the Human Research Ethics Committees at Monash Health (NMA/ERM Reference Number: 58533 and Monash Health Ref: RES-19-0000755L−58533) and The Alfred Health (Project Number: 62256 and Local Reference: Project 252/20). The patients/participants provided their written informed consent to participate in this study.

## Author Contributions

VT drafted the manuscript and developed the Monash Partners Capacity Building Framework for Pressure Injury Surveillance and Prevention with support and guidance from CW, AJ, and HT. All the authors critically reviewed and contributed to the individual parts of the manuscript and approved the final version.

## Funding

This project was supported by the Australian Government's Medical Research Future Fund (MRFF) as part of the Rapid Applied Research Translation program through Monash Partners.

## Conflict of Interest

The authors declare that the research was conducted in the absence of any commercial or financial relationships that could be construed as a potential conflict of interest.

## Publisher's Note

All claims expressed in this article are solely those of the authors and do not necessarily represent those of their affiliated organizations, or those of the publisher, the editors and the reviewers. Any product that may be evaluated in this article, or claim that may be made by its manufacturer, is not guaranteed or endorsed by the publisher.
